# Gut microbiome homeostasis and the future of probiotics in cancer immunotherapy

**DOI:** 10.3389/fimmu.2023.1114499

**Published:** 2023-05-16

**Authors:** Ankita Singh, Sharon Grace Alexander, Sunil Martin

**Affiliations:** Synthetic Immunology Laboratory, Cancer Research Division, Rajiv Gandhi Centre for Biotechnology, Thiruvananthapuram, Kerala, India

**Keywords:** gut microbiome, cancer immunotherapy, check point blockade inhibitors, stem cell transplantation, CAR T cells

## Abstract

The gut microbiome has an impact on cancer immune surveillance and immunotherapy, with recent studies showing categorical differences between immunotherapy-sensitive and immunotherapy-resistant cancer patient cohorts. Although probiotics are traditionally being supplemented to promote treatments or sustain therapeutic benefits; the FDA has not approved any for use with immunotherapy. The first step in developing probiotics for immunotherapy is identifying helpful or harmful bacteria down to the strain level. The gut microbiome’s heterogeneity before and during treatment is also being investigated to determine microbial strains that are important for immunotherapy. Moreover, Dietary fiber intake, prebiotic supplementation and fecal microbiota transplantation (FMT) were found to enhance intratumoral CD8+ T cell to T-reg ratio in the clinics. The possibility of probiotic immunotherapy as a “living adjuvant” to CAR treatment and checkpoint blockade resistance is actively being investigated.

## Introduction

The direct and indirect effect of intestinal microflora on immune response and host physiology due to a gut-organ axis is transforming cancer immunology (1, 2). Probiotics are therapeutic formulations with which beneficial microbes are identified, multiplied as such, or genetically altered to provide health benefits or augment standard-of-care therapies (3). In this context, we review the role of microbiota in cancer immunotherapy considering the recent impactful findings in Hematopoietic Stem Cell Transplantation (HSCT), Immune Checkpoint Blockade (ICB) and Chimeric Antigen Receptor (CAR) therapy to get the pulse of the field and to understand the problems and prospects of probiotics as therapeutics in the clinics. the composition of the gut microbiome is reported to impact the clinical results of CAR-T cell therapy for lymphoma according to recent investigations.

## Probiotics in shaping the fate of immunotherapy

**Figure 1:**
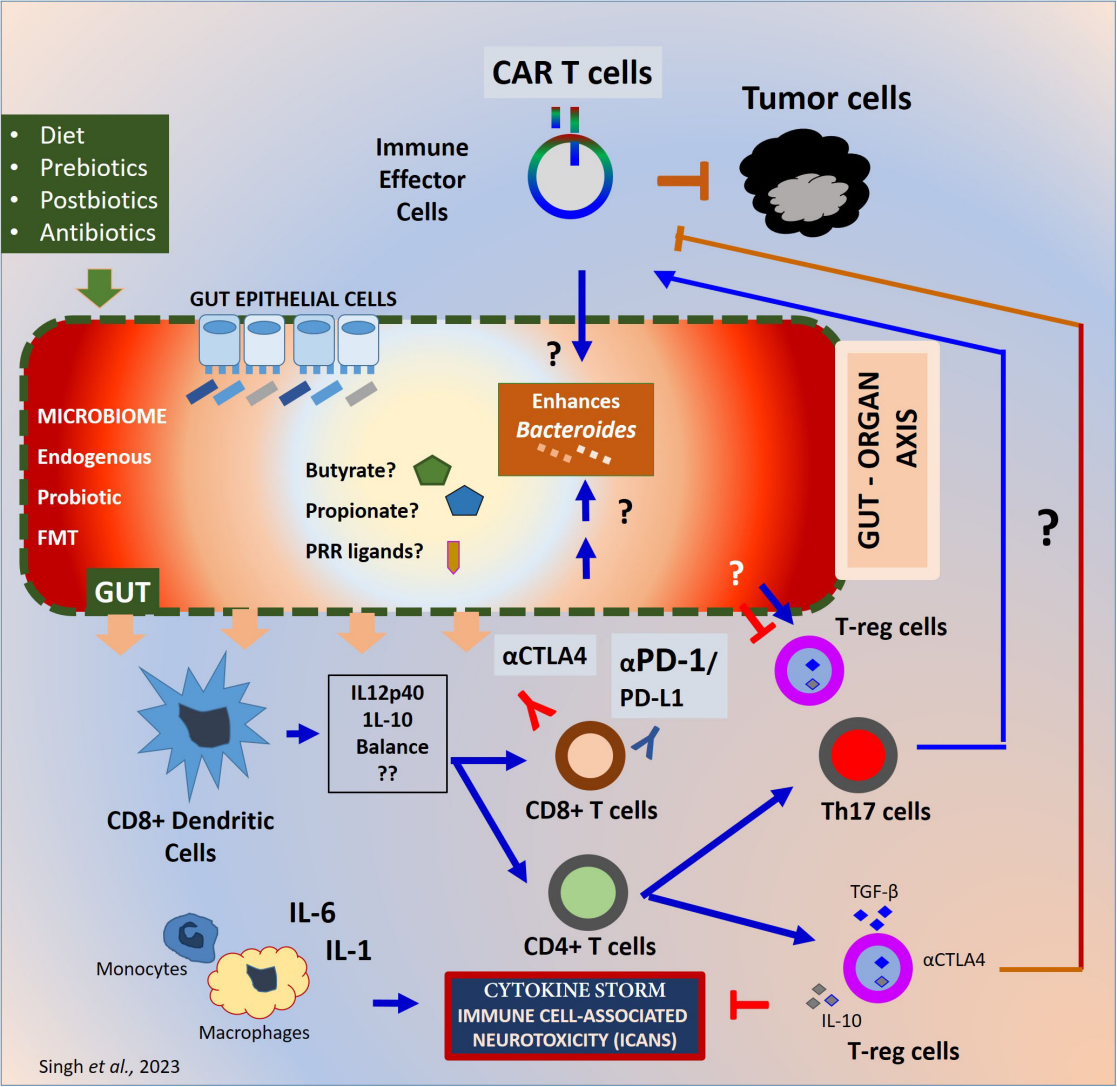
Mechanisms of gut microbiome regulation of cancer immunotherapy response. Mechanisms of gut microbiome effect on immunotherapy. The gut microbiome from endogenous or probiotic or FMT is known to maintain the barrier homeostasis and the integrity of the gut epithelial barrier. The prebiotics in the form of diet and antibiotic exposure before or during the treatment may differentially influence the diversity and homeostasis of the microbiome. The gut microbiome generates PRR ligands or metabolites which can alter the innate and adaptive immune response at the local and systemic levels. Depending upon the inflammatory milieu, Dendritic cells can be activated to alter the IL-12p40 to IL-10 ratio in presence of the microbes or microbial product. Such alteration can differentially affect the inflammatory balance. The CD8+ T cells primed can eventually trigger the antitumor immune response whereas the CD4+ T cells can help adaptive immune response by cytokine secretion and surface stimulation. The CD4+ T cells can differentiate into immunosuppressive T-reg cells and Th17 cells. T-reg cells can block exaggerated immune responses generated during immunotherapy and protect the host from inflammatory damage. Whereas T-reg cells prevent the immune response against tumor cells as well. The inflammatory cytokines such as IFNγ secreted during CAR Therapy or checkpoint blockade therapy with CTLA-4 or PD-1 blocking antibodies can generate an exaggerated inflammatory cytokine milieu. CRS or ICANS can result especially with the living-drugs such as CAR-engineered T cells negatively impacting the efficacy and patient survival. Although the exact mechanisms are unknown. PRR, Pattern recognition receptors; CRS, Cytokine release Syndrome; ICAN, Immune Effector Cell Associated cytotoxicity; FMT, Fecal Microbiota Transplant.

Preclinical studies on cancer immunosurveillance have led to translational research on immunotherapy that is now being used alongside standard cancer treatments such as surgery, radiotherapy, chemotherapy, and targeted antibody therapy (4, 5). Advances in antibiotic therapeutics such as; 16s rRNA sequencing, metagenomics, germ-free mice technology, fecal microbiota transplantation (FMT), prebiotics, synbiotics, and post-biotics has allowed researchers to explore the impact of host-microbiome on cancer immunotherapy (6, 7). Computational tools are improving our understanding of host-microbial interactions, aiding researchers in developing effective therapeutic solutions. Immune checkpoint blockade (ICB), Hematopoietic stem cell transplantation (HSCT), TCR and CAR-engineered T cells and oncolytic viral therapy are some of the major advancements that is transforming immuno-oncology. Augmenting beneficial gut microbiome was found to enhance the safety and efficacy of almost all immunotherapies that target cancer (8–11). In **Figure 1**, mechanisms of microbial regulation of cancer immunotherapy response and potential therapeutic targets are illustrated.

## Immune checkpoint blockade therapies

Although promising, the response rate of ICB needs to improve from the current value of 15-20% (12). In addition to other standard-of-care therapeutics, anti-cytotoxic T lymphocyte antigen-4 (CTLA-4), anti-programmed death protein-1 (PD-1), and anti-programmed death-ligand 1 (PD-L1) antibodies are now practiced in clinics. (13). Despite its effectiveness, one of the significant drawbacks of this method is the occurrence of immune-related adverse events, including inflammatory colitis (14). Intriguingly, recent reports demonstrated the effect of gut microbial composition on the ICB treatment efficacy and incidence of colitis (15–17). A retrospective study in cancer patients indicates that the impact of specific bacterial species on ICB efficacy varies between treatment regimens (Vancheswaran 18). The study suggests that an overabundance of *Bacteroidetes* species may lead to a poor antitumor response in PD-1-targeting ICB therapy, whereas the presence of Firmicutes species may improve efficacy (19).

Fecal microbiota transplantation (FMT) in combination with checkpoint inhibitors can reprogram the tumor microenvironment and activate host immunity with favorable changes in immune cell infiltrates in patients with prostate cancer, melanoma, and gastrointestinal cancer (9, 20–22). In a phase I trial that combined anti-PD-1 therapy with fecal microbiota transplantation (FMT) to treat metastatic melanoma, three out of 10 patients responded to the therapy response (CR) (NCT03353402), with one patient experiencing a complete response. Diwakar et al. observed in a clinical study that FMT can help to overcome resistance to anti-PD-1 therapy (pembrolizumab), resulting in clinical benefits in 6 out of 15 patients (23, 24). Probiotics and/or bacteria consortia containing live bacteria such as *Bifidobacteria*, *Lactobacillus, Propionibacterium*, and *Streptococcus thermophilus* combined with monoclonal antibodies (anti-PD-1 and anti-CTLA-4 antibodies) can significantly improve the outcomes of patients with refractory cancer (25). Probiotics have been found to improve proinflammatory cytokines and reduce IL-8 positive myeloid cells in patients. Clinical trials have shown that the tumor reduction due to probiotics is comparable to that of PDL-1 blockade, indicating that probiotics may have potential as cancer therapeutics (8). The study also revealed that lowering the fiber content can decrease the production of IFNγ and cytotoxic T-cell response (26). Consequently, the results suggest that incorporating probiotic supplementation with ICB therapy can offer substantial benefits (27, 28).

## Adoptive immunotherapy

### Adoptive immunotherapy hematopoietic stem cell transplantations

Hematopoietic stem cell transplantation (HSCT) is an intervention for hematologic disorders and malignancies involving transplantation of multipotent stem cells from patients or healthy individuals (allo-HSCT) (29–31). The gut microbiome has a significant impact on the immune reconstitution, therapeutic outcomes, and side effects, such as infections, of hematopoietic stem cell transplantation (HSCT) (32). Gut microbiome imbalance often occurs in HSCT due to transplantation procedures (33, 34). In general, maintaining the Bacteroides is considered beneficial for treating acute GvHD. Broad-spectrum antibiotics and altered diet during the treatment often directly impact the gut microbial community depleting the *Bacteroidetes* species (35). Since microbial metabolites maintain the gut epithelial integrity and palliate graft versus host disease (GvHD), disruption of microbes can have a potential impact on the therapeutic outcome of HSCT. However, HSCT-associated drugs can cause dysbiosis and related complications (36–38). Studies have shown that a higher diversity of gut microbiome is associated with improved clinical outcomes after HSCT. Nonetheless, the mechanism behind this impact can vary based on the model and donors (39–41).

Doki et al. in a clinical trial observed that the ratio of *Firmicutes* to *Bacteroides* is high in patients with acute GvHD (42). In a human study co-relating microbial diversity and GvHD; Treg/Th17 ratio was positively co-related with Clostridia (*Lachnospiraceae* and *Ruminococcaceae*) and negatively with *Gammaproteobacteria* (43). The addition of microbes and their metabolic product appears to ameliorate acute GvHD and maintain a tolerogenic state apart from maintaining the integrity of the gut epithelia (44). Further research is needed to understand the microbial taxonomy, microbial antigens, and metabolism.

In patients who received all-HSCT, vancomycin prophylaxis was given to prevent *Clostridium difficile* infection, which led to elevated IL-12p40 levels compared to historical controls. Although the gut microbiome’s impact on HSCT is known, the mechanism may vary depending on the donors and models used (45). Hence, conducting a comprehensive study can help reveal the exact immunological mechanisms by which the gut microbiome affects HSCT.

### Adoptive transfer of therapeutic T cells

In a distinct study, researchers found that the effectiveness of adoptive cell therapy after radiation is affected by a temporary infection of the gut microbiome and the activation of dendritic cells in the mesenteric lymph node (46). In a murine model of lung and cervical cancer, it has been demonstrated that higher *Bacteroidales* than *Bacteroidetes* were associated with reduced tumor burden after the adoptive transfer of TCR-engineered T cells. The response rate to adoptive T cell transfer varied among animal cohorts with different gut microbiota. Neomycin/metronidazole treatment did not affect T cell transfer, but vancomycin or fecal microbiota transplantation resulted in reduced tumor volume in mice treated with TCR-engineered T cells. This improvement was associated with increased expression of Granzyme B, Perforin 1, IL-12, and IFNγ, indicating a typical Tc1 profile. Mechanistically, IL-12 secreted from the systemic CD8α+DCs was elevated in both these treatments and IL-12 neutralization reduced the type 1 T cells response (47). That being the case, the gut microbial composition can impact tumor infiltration and *in vivo* expansion after T cell transfer.

### CAR therapy

CAR therapy is an FDA-approved cell therapy for multiple blood malignancies (48, 49). Although CAR therapy is successful, a sizable number of patients are either ineligible or unresponsive to the therapy. After CAR therapy, around 60% of patients relapse with up to 80% of patients manifesting toxicities including cytokine release syndrome (CRS) and neurotoxicity (50).

The gut microbiome can be a potential factor contributing to the differential effect of CAR therapy across patients. Researchers and clinicians have long sought to uncover the connection between gut microbial taxa and the outcome of CAR therapy. Memorial Sloan Kettering Cancer Center (MSKCC) conducted a comprehensive study to investigate the correlation between gut microbial compositions prior to CAR therapy and complete response or toxicity (51). To monitor the toxicity, the clinical response was graded as either complete response (CR) or no CR apart from the grades of (CRS) or neurotoxicity. Despite variations in patient cohorts’ treatment history, pre-treatment samples of patients who achieved complete remission (CR) were found to have high abundance of *Scilliospiraceae*, *Ruminococcacaeae*, and *Lachnospiraceae*. Meanwhile, patients who did not achieve CR showed enrichment of *Peptostreptococcaceae*, which are known for synthesizing B vitamins. This is perhaps the first study that systematically co-related microbiome and CAR T cell therapy outcome (52). However, a direct link between the outcome of CAR therapy and individual bacterial species has yet to be established. Additional studies may reveal how the enrichment of vitamin B synthesizing genes and clinical response is related to CAR therapy. The relationship between the efficacy and toxicity of CD19 CAR T-cell therapy and the gut microbiome can be studied by evaluating prior exposure to antibiotics in patients. A larger retrospective study by researchers at MSKCC showed a clear association between specific antibiotic use and therapeutic outcome (53). An increased chance for Immune Effector Cell Associated Neurotoxicity (ICANS) was observed in patients exposed to piperacillin/tazobactam, meropenem, and imipenem/cilastatin with B-cell acute lymphoblastic lymphoma (B-ALL) or non-Hodgkin’s lymphoma (NHL).

The presence of high Bacteroides species in fecal microbiomes was found to be associated with predicted toxicity during CAR therapy. *Firmicutes* were associated with reduced toxicity as they produce short-chain fatty acid butyrate as metabolites. A higher butyrate concentration enhances IFNγ expression through T-bet whereas a lower butyrate concentration enhances T-reg cells secreting IL-10 (54). The exposure to certain antibiotics, including piperacillin/tazobactam, meropenem, or imipenem, four weeks prior to CD19 CAR T cell treatment was found to be associated with poor survival outcomes (OS and PFS) and increased toxicity, particularly neurotoxicity, in patients with ALL and NHL. The study suggests that the brain-gut axis may have a role in neurotoxicity during CAR T cell therapy and highlights the potential impact of antibiotic use on gut microbiota and treatment outcomes. However, due to the small sample size and lack of mechanistic insights, further research is needed to establish a causal relationship between specific microbial species and CAR therapy response.

Similar to the 2019 study on HSCT from MSKCC, the effectiveness of CAR therapy was linked to high levels of *Ruminococcus* species and *Lachnospiraceae* in complete responders. Interestingly, these high levels were also associated with an increased ratio of Treg/TH17 cells, indicating their role in balancing anti-inflammatory and pro-inflammatory responses (54). *Ruminococcaceae* is implicated in enhanced immune response involving monocytes, neutrophils, and lymphocytes (55). In a recently reported multicentric study published in Nature Communications, the authors addressed the microbiome question at various phases of CAR therapy in relapsed/refractory non-Hodgkin lymphoma, acute lymphoblastic leukemia, and multiple myeloma (56). In this clinical trial (ChiCTR1800017404) the differential gut microbiome among patients is indicative of clinical response to CAR therapy. The gut microbiome may have a significant impact on the response to CAR therapy in multiple myeloma, with some bacterial species being associated with response and others with CRS.

The researchers in the study separated patients into groups based on the type of antibiotics they were given prior to CAR-T cell therapy. They then analyzed the impact of the gut microbiome composition on treatment outcomes. Patients who received high-risk antibiotics which target anaerobic commensals and have broad-spectrum activity, in the three weeks leading up to CAR-T cell therapy had higher rates of lymphoma progression, lower progression-free survival, and lower overall survival compared to patients who had low-risk or no antibiotic exposure (57). To ensure that the negative impact of high-risk antibiotics on CAR-T cell therapy outcomes was not due to confounding factors, the researchers analyzed the gut microbiomes of patients who had not received these antibiotics. They identified certain microbiome characteristics that were associated with positive treatment outcomes in lymphoma patients from different parts of the world, suggesting that targeting these features could improve CAR-T cell therapy efficacy. Further studies are needed to fully understand the role of the gut microbiome in CAR therapy response, but the knowledge gained may lead to interventions that involve administering specific beneficial gut bacteria along with CAR therapy.

### Use of probiotics in cancer immunotherapy: challenges

Probiotics, specifically *Lactobacillus* and *Bifidobacterium*, have a long history of safety and are considered generally recognized as safe (GRAS). They have shown promise in the prevention and treatment of cancer in various cancer types. However, there are still several hurdles to overcome before translating probiotics into effective cancer treatment options.

Clinical studies, industrial production, and commercialization have been inconsistent in their outcomes across various health problems. This could be due to variabilities in the experimental system, trial design and lack of analytical rigor. For instance, a literature review from 1990 to 2017 showed that the effect of probiotics on gut microbiome composition is temporary, and its impact on lipid profile is not consistent (58). The variability of the bacterial species across healthy individuals as well as patients apart from complicated crosstalk between microbial spp. makes it hard to associate microbiome diversity with the patient response (20, 59). The beneficial effects of probiotics are specific to certain strains, and therefore, it is necessary to identify the beneficial probiotics for each type of cancer precisely (60). Towards this effect, a human database may be built to catalog the favorable strains for therapy and prevention. Additionally, a therapeutic approach should be developed to eliminate harmful microbes, thereby promoting a microbial balance that benefits the host. During treatment, it’s crucial to keep track of the clinical response as a function of the gut microbiome.

Establishing the gut microbiome as a “living supplement” in the gut is a significant challenge. Crossing the gastrointestinal tract with proteolytic enzymes and sharp pH variation is a major challenge in probiotic therapy (3). In contrast, the digestive capacity and the uptake of the individual also need to be considered while devising the probiotic therapy (61). Additionally, metagenomics analysis of the taxonomic and functional relationship between resident and beneficial bacteria is essential to precisely dope probiotics into cancer therapy regimens.

The long-term therapeutic effect of probiotics has not yet been formally established and the mass production and commercialization of probiotics for cancer treatment are complicated due to safety concerns for immunodeficient or immunocompromised cancer patients (3). Antibiotic resistance genes in probiotics remain to be a concern as well. *In vivo* animal studies, whether preventive or therapeutic, mostly focus on colorectal and breast cancers (62, 63).

One of the imperatives in the arena is to elucidate the immunological mechanisms of how probiotics act or function. Importantly, the anaerobic culture conditions for most of the probiotics possess challenges in immunological explorations (64). There are challenges associated with modifying probiotics’ DNA using recombinant technology, which is essential to enhance their therapeutic potential. To address this, regulatory and biosafety guidelines for the genetic modification of probiotics need to be established (65). The use of a biomarker to predict the success of immune therapy is feasible given the wide variety of microbial taxa in clinical trials. Studies suggest that the gut microbiome may affect the response to CAR-T cell therapy, but it is not yet clear if this association is causal. Future research should include mechanistic and interventional studies to explore the microbiome’s role in lymphoma responses and improve treatment outcomes (57). The success of fecal microbiota transplantation in other contexts provides evidence that the microbiome can be targeted for therapeutic intervention (69), Davar et al. (23). Similar to the impactful findings in ICB or FMT, combining specific microbial species with CAR therapy may be a potential strategy to decrease toxicity and improve efficacy.

### Prebiotics, post-biotics and synbiotics in cancer immunotherapy

Prebiotics are selective nondigestible food components that stimulate the expansion of selective gut microbes (66, 67) for example, oligomers of fructose and galactose, and inulin. Some human studies have shown that prebiotics can help restore gut microbial diversity and assist in cancer therapy. This prebiotics have varying effects, including microbe-independent immunostimulatory activity. Synbiotics combine specific prebiotics with probiotics to promote their growth. *Lactobacillus rhamnosus* and *Bifidobacterium lactis*, when combined with prebiotics, have been found to inhibit colon cancer (68). To avoid infection risk in cancer patients with weakened immune systems, beneficial substances produced by microbes or prebiotics can be administered instead of probiotics. Microbe-free formulations of probiotics may also be used. Immunotherapy can be combined with prebiotics, probiotics, postbiotics, and symbiotics in upcoming immune-oncology trials (69). The completed clinical trials testing the effect of probiotics on cancer immunotherapy is listed in **Table 1**.

**Table 1:** Clinical trials with the probiotics in cancer therapy.

S.No.	Conditions	Probiotic tested clinical trial	Phase	Trial number
1.	Colorectal Cancer	HEXBIO, Bifidobacterium longum BB536, Lactobacillus johnsonii LA1 (LA1), Saccharomyces boulardii, Colon DophilusTM , Lactobacillus Rhamnosus supplementation.	II, III, IV	NCT03782428, NCT00936572 NCT01895530 ,NCT01609660 NCT01410955 ,NCT00197873
2.	Colorectal Neoplasm	Synbiotic Forte™, "IONIA" Pharmaceuticals, Athens, Greece)	NA	NCT01479907
3.	Rectal Cancer	Lactobacillus plantarum HEAL 19	NA	NCT03420443
4.	Liver Cancer	Lactobacillus rhamnosus Probio-M9	NA	NCT05032014
5.	Breast Cancer	Primal Defense Ultra® Probiotic Formula, lyophilised Lactobacillus strains, Saccharomyces boulardii, Lactobacillus spp., Bacillus subtilis, Bifidobacterium spp., Lactobacillus rhamnosus GR-1 and Lactobacillus reuteri RC-14.	NA	NCT03358511, NCT01723592 NCT04784182, NCT03290651
6.	Hematologic Cancer or Myelodysplastic Syndrome	Culturelle DS (Lactobacillus GG)	NA	NCT00946283
7.	Lung Cancer	Clostridium butyricum	I	NCT02771470,
8.	Sigmoid Colon Cancer	Mechnicov’s probiotics	NA	NCT03531606
9.	Colon Cancer	Bifidobacterium lactis Bl-04 (ATCC SD5219), Lactobacillus acidophilus NCFM (ATCC 700396).	NA	NCT03072641
10.	Gastrointestinal Cancer	Lactobacilli strains, Bifidum strains, Streptococcus thermophilus	NA	NCT03704727
11.	Head and Neck Cancer	Arkoprobiotics® Defenses	NA	NCT03720015
12.	HPV infection	Lactobacillus rhamnosus BMX 54, Lactobacillus reuteri RC-14, Lactobacillus rhamnosus GR-1	NA	NCT05109533
13.	Hepatocellular Carcinoma	Lactibiane TOLERANCE	NA	NCT02021253

## Discussion

Decades of research in probiotics ever since the time of Metchnikoff led to the establishment of industrial production of prophylactic and therapeutic probiotics. The therapeutic potential, however, remains largely unproven. To address this, preclinical and clinical trial protocols for probiotic cancer immunotherapy need to be standardized to ensure high quality and rigor. Next-generation probiotic formulations, genetically engineered to target specific species for individual cancer types, may overcome current limitations. While the idea of using FMT or other interventions to increase the abundance of beneficial gut microbiota is promising, there are logistical challenges to overcome. Traditional FMT has donor screening and donation-related obstacles, as well as a small infection risk. New approaches to transfer specific bacterial strains are still being developed. However, the study provides valuable insights into the influence of the gut microbiome on immunotherapies like CAR-T cells and provides a foundation for future interventional approaches. Large-scale investigations in the future, utilizing multi-omic read-outs to study the long-term effects of probiotic immunotherapy, can help identify the most effective strategies for cancer treatment and prevention. With the advancements in genome editing, probiotic immunotherapy has the potential to tackle tumor resistance in multiple malignancies. Further clinical studies along these lines are awaited.

## Author contributions

AS: Collected literature, extracted the data, prepared the table, wrote the manuscript SM: Conceptualized the theme, extracted the data, prepared the table, wrote the manuscript, prepared the figures. All authors contributed to the article and approved the submitted version.
